# AKT inhibition mitigates terminal differentiation and preserves central memory phenotype of CD8 T cells

**DOI:** 10.1186/2051-1426-2-S3-P93

**Published:** 2014-11-06

**Authors:** Rasha Abu Eid, Kevin Friedman, Mikayel Mkrtichyan, Samir N Khleif

**Affiliations:** 1 Georgia Regents University Cancer Center, Augusta, GA, USA; 2 bluebird Bio, Cambridge, MA, USA

## Introduction

CD8 T cell response comprises effector and memory T cells. Effector CD8 T cells become terminally differentiated and are eliminated by apoptosis. Memory CD8 T cells encompass central (T_CM_) and effector memory T cells (T_EM_). T_CM _display a higher proliferative ability, express a higher level of CD62L and are superior in their protection against viral and bacterial challenges and mediation of anti-tumor immunity when compared to T_EM_. The differentiation of CD8 T cell is thought to be coordinated by the PI3K/Akt pathway.

## Methods

The effect of *in vitro *Akt inhibition using the pan Akt inhibitors MK-2206 and AZD5363 on the differentiation and proliferation of CD8 T cells was examined. Cell proliferation, expansion, cytokine production and phenotype were assessed in antigen specific CD8 T cells.

## Results

We found that the inhibition of Akt leads to a significant enhancement of the proliferative potential of CD8 T cells, and to prolongation of their survival upon TCR re-stimulation. Furthermore, we found that Akt inhibition leads to increase in IL-2 secretion, a marker of cells with high proliferative ability. We further identified that Akt inhibition preserved the T_CM _phenotype by conserving a higher percentage of (CD44^HI ^CD62L^HI^) expressing T cells. These cells also displayed a higher level of CD127 and a lower level of the exhaustion marker KLRG-1 reflecting their increased expansion ability and longevity due to Akt inhibition. Additionally, we found that Akt inhibition also resulted in the preservation of a significantly higher percentage of naïve CD8 T cells (CD44^LO ^CD62L^HI^) when compared to the non-treated cells.

## Conclusion

Here, we show that Akt inhibition preserves the central memory phenotype of CD8 T cells thus enhancing their proliferation, survival and cytokine production. Furthermore, Akt inhibition results in the conservation of a reservoir of naïve CD8 T cells. Both naïve and T_CM _CD8 T cells are superior mediators of anti-tumor immunity when compared to effector or T_EM _cells. These findings strongly suggest the utility of using Akt inhibitors to modulate the immune response as part of cancer immune therapy.

